# Vertebrae and intervertebral discs segmentation using deep learning-based model in disability analysis

**DOI:** 10.3389/fmed.2026.1723191

**Published:** 2026-02-25

**Authors:** Nizar Alsharif, Rajit Nair, Theyazn H. H. Aldhyani, Nesren S. Farhah, Sultan Ahmad, Abdullah H. Al-Nefaie

**Affiliations:** 1 King Salman Center for Disability Research, Riyadh, Saudi Arabia; 2 Department of Computer Engineering and Science, Al-Baha University, Al-Baha, Saudi Arabia; 3 VIT Bhopal University, Bhopal, India; 4 Applied College, King Faisal University, Al-Ahsa, Saudi Arabia; 5 Department of Health Informatics, College of Health Science, Saudi Electronic University, Riyadh, Saudi Arabia; 6 Department of Computer Science, College of Computer Engineering and Sciences, Prince Sattam Bin Abdulaziz University, Al-Kharj, Saudi Arabia; 7 School of Computer Science and Engineering, Lovely Professional University, Phagwara, India

**Keywords:** convolution neural network, deep learning, graph convolutional segmentation network, magnetic resonance imaging, vertebrae and intervertebral discs

## Abstract

Segmentation of vertebrae and intervertebral discs (IVDs) is a cornerstone of the diagnosis and treatment of disorders affecting the spine. Yet, most methodologies, especially CNN-based, mostly treat vertebrae and discs independently, missing out on the potential of their anatomical relationships. To fill this gap, we present a two-stage deep learning framework that incorporates structural dependency modeling to automate spine segmentation in T2-weighted MR images. In the framework, the components of the spine are modeled as nodes of a graph, with anatomical relationships stored in the system’s adjacency matrix. A 3D Graph Convolutional Segmentation Network (GCSN) is first used to perform coarse multi-class segmentation, leveraging the relationships between vertebrae and discs. Then, a 2D ResNet refinement network is used to enhance boundary resolution. The model was tested on volumetric MR data of 218 subjects. The average Dice similarity coefficient (DSC) across 10 vertebrae was 87.32%, 87.78% across 9 intervertebral discs, and 87.49% across 19 structures in the spinal column, showing exemplary segmentation performance. The result shows that the segmentation consistency and accuracy have improved significantly due to the use of the anatomical dependencies through the graph-based learning approach. The proposed system provides a safe and highly effective automated system for parsing the spine and can be clinically used for diagnosing and planning the treatments for spinal disorders.

## 1 Introduction

**FIGURE 1:**
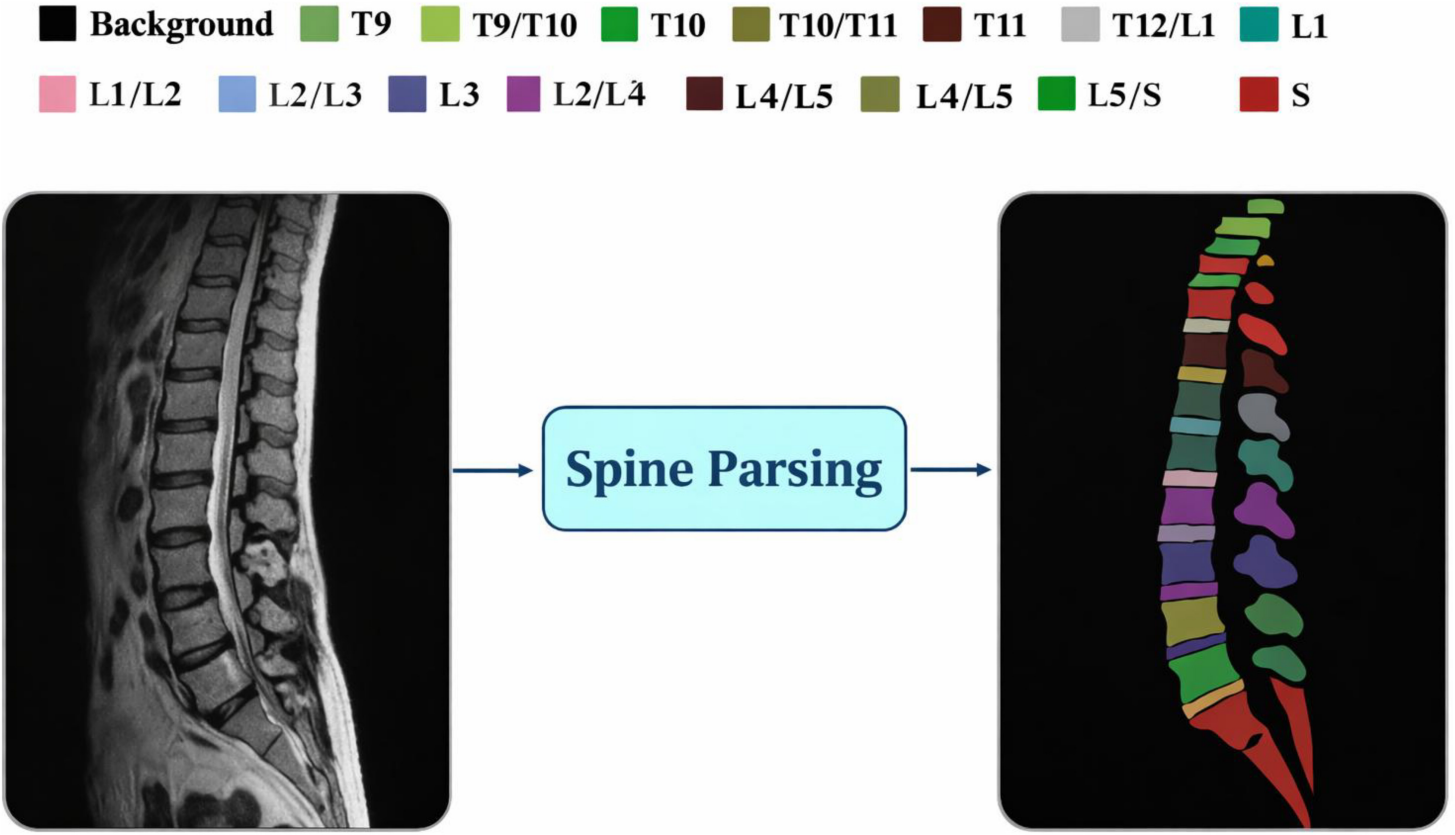
Illustration of spine parsing, showing the division of the spinal column into thoracic (T), lumbar (L), and sacral (S) regions for volumetric MR image segmentation.

The diagnosis, treatment planning, and image-guided intervention for spinal disorders rely on accurately segmenting vertebrae and intervertebral discs (IVDs) from medical images. Precise vertebral structure and IVD localization is vital for applications like lumbar radiofrequency ablation (RFA), disc herniation assessment, spinal degeneration analysis, and surgical navigation. However, while computed tomography (CT) gives detailed visuals of bony anatomy, it presents limited contrast for soft tissues like intervertebral discs. On the other hand, magnetic resonance (MR) imaging gives a detailed soft tissue contrast which catapults it as the better option for thorough spinal analysis. As a result, modern spine-related clinical workflows and advanced computer-assisted interventions need automated spine parsing from volumetric MR images (1–3). Lumbar decoding, or cross edge detection of the spine, has various orthopedic use cases, especially for vertebrae body imaging and intervertebral disc (IVD) detection and analysis. These use cases are for the diagnosis and treatment, including mini-invasive techniques like percutaneous procedures, for numerous spinal disorders. Besides the communication within the treatment, such programs facilitate assessment and communication of therapeutic plans, which are all performed per the defined standard operating procedures. A frequent treatment of this nature is lumbar radiofrequency ablation (RFA). In this approach, the injector’s tip is usually steered to the target through the use of Computed Tomography (CT) imaging (4). Also, like lumbar RFA, seeing the intervertebral discs is very important for the successful localization of herniated discs. However, relied solely on CT scanning, the IVDs can be represented neither clearly nor reliably due to the soft tissue contrast. This issue can be solved by CT and MR image fusion, since MR imaging has the IVDs contrast. The spine comprises both rigid (vertebrae) and non-rigid (soft tissue and discs) structures. Therefore, global rigid or affine registration methods are inappropriate for the MR-CT spine image registration, as shown in Figure 1. The vertebrae are the only rigid structures of the spinal column, while the other areas require elastic deformation. Therefore, a hybrid registration approach is viable, where rigid registration can be applied to the vertebrae and elastic registration to the surrounding soft tissues (5, 6). For this approach, precise vertebral segmentation is needed to assist in identifying and defining the rigid elements during the image registration process. Therefore, spine parsing in volumetric MR images is vital for effective lumbar RFA planning and guidance.

**FIGURE 2:**
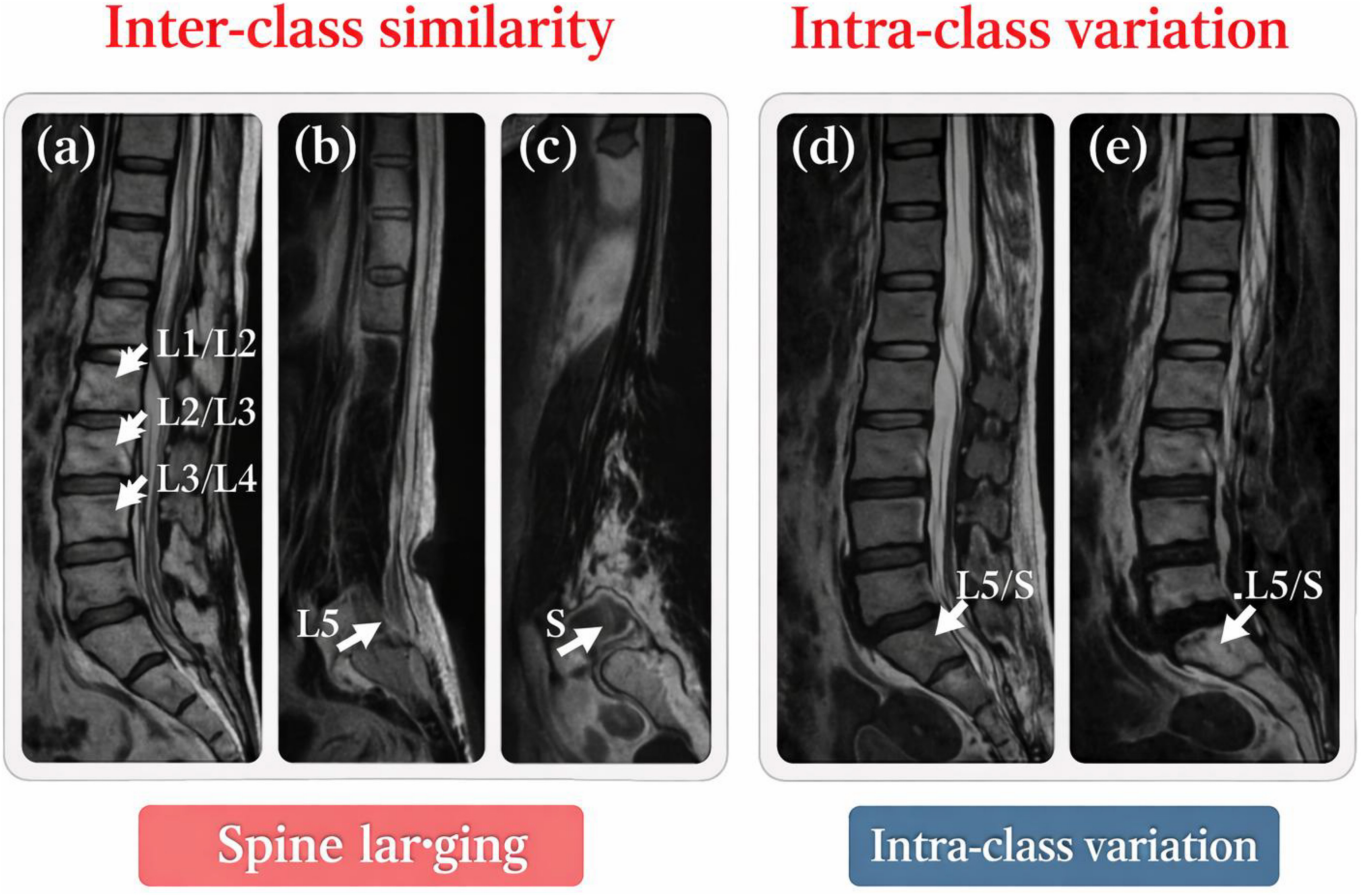
Challenges in spine parsing: **(a)** overlap between adjacent classes, **(b,c)** high inter-class similarity, and **(d,e)** large intra-class variability among intervertebral discs.

Figures 2b,c illustrate the capacity to differentiate among various patients’ intervertebral discs (IVDs) at discrete anatomical classes. Figure 2a shows a case where the patient has her/his spinal elements, particularly in the lumbosacral area, very closely positioned. In contrast, Figures 2b,c show spine columns of two different patients. Because the slices are aligned along the spine, these images demonstrate more about the likenesses and contrasts among patients. Such likenesses are a result of the discs that lie between neighboring vertebrae, which are prone to the same degree of degeneration and damage. In the axial slices of two different patients, the L5 vertebra in Figure 2b and the sacrum (S) in Figure 2c appear very much the same. Segmentation confusion is primarily a result of the inter-class likeness, which is most noticeable in 2D projections. In contrast, the intra-class variation demonstrated in Figure 2d, shows IVDs from patients with the same underlying spinal pathology, yet they are significantly different from one another. Such variation can be explained by the diverse morphologies of spinal disorders and varying disease progression (7, 8). Due to this, IVDs can be difficult to tell apart from one another. Focusing on L5/S intervertebral discs of two different patients with the same clinical diagnosis of lumbar disc herniation, Figures 2d,e show examples of differing disc structures despite the same clinical etiology. From a computational standpoint, a densely connected 3D model of the spine requires a considerable amount of computational power and memory. This is because of the large size and complex structures that make up the spine. Additionally, modeling the spine as completely rigid can create modeling errors, as spinal motion and deformation are not uniform throughout all the regions especially with the presence of external support or fixation.

Most recent spine segmentation methods that utilize backbone deep learning frameworks have been achieving reasonable results, however, most of the Convolutional Neural Network architectures (CNNs) consider vertebrae and the Intervertebral discs (IVDs) as separate entities. Such predictions are done pixel-wise and do not account for the significant anatomical dependencies that exist within the spinal column. Each vertebra is structurally and functionally connected to the vertebrae and corresponding IVDs that are spatially positioned next to each other. Because of this, conventional CNNs struggle with ambiguity of labels, confusion between classes, and propagation of errors within a given class. This is especially true when the class contains sub-anatomically similar structures like the lumbosacral junction and the areas with degenerated disc spaces (9–11). When utilizing graph-based semantic modeling, spinal structures as nodes in a graph attain a methodology that addresses the anatomical adjacency and relations that are defined and structurally encoded with edges. When graph-based methods and volumetric feature learning are combined, the ability to make a portion of a vertebral disc globally consistent, as opposed to locally consistent, can be done with relational information that flows through. In establishing dependencies, a model has been created that is ideal for parsing the spine in that defined structure has been created along with the relationships that are ordered in such a way that the segmentation of the structure is clinically reliable. This has been especially true when the segmentation has been clinically reliable (12–14). Spine parsing has recently attracted significant research attention, particularly due to its importance in generating accurate semantic representations of spinal images. Ongoing efforts focus on the segmentation of vertebrae and intervertebral discs (IVDs), which are typically treated as separate anatomical entities. Broadly, two major approaches exist for vertebral and IVD segmentation: traditional image processing–based methods and deep learning–based techniques. Both approaches have demonstrated validity, although traditional methods have historically been more prevalent. Conventional approaches often achieve optimal performance by integrating multiple techniques, such as image registration and label fusion across several atlases. For example, a multi-atlas–based method for segmenting IVDs in volumetric MR images has been proposed in Li et al. (15) and Sekuboyina et al. (16) and is discussed in greater detail later in this section. The core idea of this approach lies in utilizing multiple atlases, each representing a distinct anatomical category. The procedure typically involves three key steps: first, registering patient images to the atlases; second, identifying and labeling spinal regions to extract the relevant volumes; and third, fusing the propagated labels to generate the final segmentation (17, 18). These methods have been applied primarily to thoracic and lumbar vertebrae using data acquired from two different imaging modalities. However, segmentation errors are more pronounced in the thoracic and lumbar regions, particularly in the lower spine, due to increased anatomical variability. The study makes three primary contributions. First, we describe a framework for spine segmentation that is guided by anatomy and models the structural dependencies of vertebrae and intervertebral discs. This is an improvement over the conventional CNN-based methods that ignore the structural relationship between these components. Second, we present a novel two-stage deep learning architecture that combines the 3D Graph Convolutional Segmentation Network (GCSN), which is memory efficient and divides the segmentation task into a global, structure-based segmentation, and a 2D ResUNet, which is a high-resolution boundary refinement segmentation, for the true achievement of segmentation in a fully anatomic and multi-class manner. Third, the framework distinguishes between each vertebra and intervertebral disc, precisely performing the anatomy-level differentiation that makes it more than other T2-weighted MR images volumetric. This makes the framework more applicable for clinical spine analysis and image-guided interventions. The remainder of this paper is organized as follows: Section 2 reviews related work; sections 3 and 4 describe the proposed methodology and present the experimental results and analysis, respectively; Section 5 provides a discussion, and Section 6 concludes the paper.

## 2 Related work

All measurements were made in millimeters. A landmark-based technique used by Neubert et al. (6) to localize intervertebral discs (IVDs) is noted. Using a self-context similarity descriptor, IVDs were modeled using a deformable framework. With this approach, 92% of the IVDs were successfully recognized. In addition, a landmark detection and deformable modeling integration framework was established. This framework was used in the further identification of fractured lumbar vertebrae by the removal of vertebral bodies of the damaged vertebrae and subsequent assessment of the spine. The initialization of deformable models can be manual (19) or automatic (20), and this choice has a significant bearing on the accuracy of segmentation. An alternative method using a combination of machine learning for the identification and segmentation of IVDs in volumetric MR images is presented (21). The center of the discs was tracked using a displacement model, and for model training and testing, the methodology was data driven in accordance with defined parameters. The final IVD segmentation was accomplished by a classifier that used visual features to analyze and classify the image voxels in the proximity of the center of the discs. Many machine learning-based approaches used voxel patch by hand features to discern various discs. These features, however, were suboptimally integrated for specific segmentation tasks. The segmentation performance and the computational efficiency was ultimately limited by the engineering of features, which is painstaking, and in the end this approach resulted in a significant reason for the performance efficiency of segmentation to be that power barrier (10).

Though vertebrae and intervertebral discs (IVDs) can lately be completely automated through deep learning, some setbacks still exist. His early techniques were multi-atlas registration and the use of deformable models, which obtained great accuracy, but at great cost because they were sensitive to the variability of structures. Then deep learning arrived, and fully convolutional networks (FCNs) become the standard. U-Net architectures and even 3D networks became the go to models for spine segmentation from MR and CT to offer improved robustness and efficiency. Later, researchers used attention mechanisms, multi-scale feature fusion, adversarial learning (GANs), and sequence models to improve contextual understanding and delineation of graph boundaries. Still, the vast majority of techniques frameworks treat vertebrae and IVDs as completely separate structures, and often assign the same class label to all the vertebrae and discs. Many techniques, or frameworks, address the spine vertebrae and IVDs as separate structures which, with the loss of class, label, and chain structure of the spine, can lead to class confusion. In cases of high inter-class similarity or severe degeneration there can be a cascading loss of class, label, and structure of the spine chain which can cause high turnover and reduce the efficacy of the technologies. Comparative to all, the new technologies model vertebrae and IVDs as a single spine dissenting structured interrelated entity through a graph-based semantic learning structure, and combines high resolution 2D with global 3D context. This design allows for the first time anatomically plausible multi-class segmentation and successfully tackles the challenges posed by previous approaches with regards to structural dependency modeling, memory efficiency and detailed anatomical differentiation.

While Fully Convolutional Networks (FCNs) and U-Net architectures are efficient in terms of computing resources at inference time, they do not provide a means of explicitly understanding the relationships between neighboring anatomical structures. This is especially important in spinal imaging, where vertebral bodies and intervertebral discs are in close proximity to each other and are dependent upon each other structurally. For instance, the L2–L3 disc is always positioned between the L2 and L3 vertebrae, with L2 positioned superior to L3, creating a defined spatial relationship. It is important to utilize such relationships to meaningfully learn to represent and differentiate between the various parts of the spine. With this in mind, Fallah et al. (22) was the first to vertebrae CT image segmentation and labeling and later described a method for spinal localization and labeling in MR images. Because of this, there has been more reliance on the segmentation and dependency aware segmentation of components of spinal images.

While GANs have proven to be helpful in refining segmentation boundaries in these types of scenarios (23), the current iterating FCN-based frameworks have not been able to overcome the cascading error challenges. In each GAN iteration, for example, if one vertebra is incorrectly localized or segmented, the resulting misprediction of all remaining vertebrae is largely due to the significant anatomical similarity amongst vertebral segments. However, unlike GANs, our framework centers on modeling vertebral interrelations in feature space, rather than in the prediction space. This approach, we posit, primarily allows for the enhanced exploitation of the spatial hierarchies of spinal structures, thereby resulting in improved segmentation of vertebrae and intervertebral discs. Cascading errors still plague progressive FCN-based frameworks, despite their ingenuity. For instance, when first vertebral bodies are incorrectly localized/segmented, subsequent vertebral bodies are frequently improperly localized/segmented because of their high anatomical resemblance. In these situations, GANs learn inter-vertebral relationships in mesos of segmentation hypothesis space and thus, can improve segmentation boundaries. However, we model inter-vertebral relationships in feature space, not prediction space. This, in fact, provides more spatial hierarchies within spinal constituents, improving segmentation of inter-vertebral discs and vertebrae. This study has been designed to answer the research questions that respond to some of the existing limitations of spine segmentation. More specifically, in this paper, the author has analyzed the degree to which segmentation accuracy and consistency improve by explicitly reasoning about the anatomical relations of vertebrae and intervertebral discs as structures that can be dependent on one another in segmentation, using graph-based learning, as opposed to treating them as independently segmentable structures in CNN-based approaches. The author also analyzed the degree to which a two-stage, memory-efficient framework designed to produce and retain coarse 3D volumes in memory, and then refine them in 2D to a high-resolution, provide volumes metric and multi-class segmentation of the spine from volumetric MR images in an anatomically consistent manner. Finally, the author explored the extent to which the labeling of vertebrae and intervertebral discs as having anatomical detail improves the robustness and reliability of such labeling in psychologically adverse conditions where inter-class similarity, structural variability, and class imbalance exist. Collectively, the four research questions frame the academic boundaries of the work and justify the proposed approach and its experimentation the most.

## 3 Proposed method

The project examined 218 T2-weighted MR images obtained from one of the local medical centers. Among these, there were only six clinically healthy subjects, and the rest had various types of spinal pathology (24). A junior specialist made the first round of manual annotations on the vertebrae and intervertebral discs (IVDs), which were then edited by a senior specialist using ITK-SNAP. These edits were then used as the ground-truth standard for spine parsing. Most novel methodological contributions for fully automated spine segmentation from volumetric MR (Magnetic Resonance) images has been achieved in this work. We first solve spine parsing as a graph-based semantic learning problem so that individual vertebrae and intervertebral discs (IVD) as nodes of a graph model, can be disconnected in a graph, and structuring an adjacency matrix so that the anatomical relationships between intervertebral discs and vertebrae can be encoded. Such an arrangement allows the proposed model to learn the global anatomical context and the inter-structural relationships that most of the standard pixel-by-pixel CNN segmentation methodologies tend to skip. Secondly, we construct a computationally economical, and a two-tiered segmentation framework involving a 3D Graph Convolutional Segmentation Network (GCSN) for volumetric coarse segmentation, and a 2D ResUNet based refinement network for the recovery of high resolution, fine anatomical boundaries. This blended 3D-2D approach is not only computationally economical, but it also global context reasoning in contrast to local boundary precision for large clinical datasets. Thirdly, our proposed framework accomplishes anatomically consistent multi-class segmentation by differentiating vertebrae and intervertebral discs (IVD) through individual vertebrae and IVD labeling. This, along with accurate structural differentiation, improves robustness in areas with high inter-class similarity or class imbalance. Overall, our framework along with its contributions, provides a broad and clinically scalable segmentation framework to spine filtering.

### 3.1 Dataset

**FIGURE 3:**
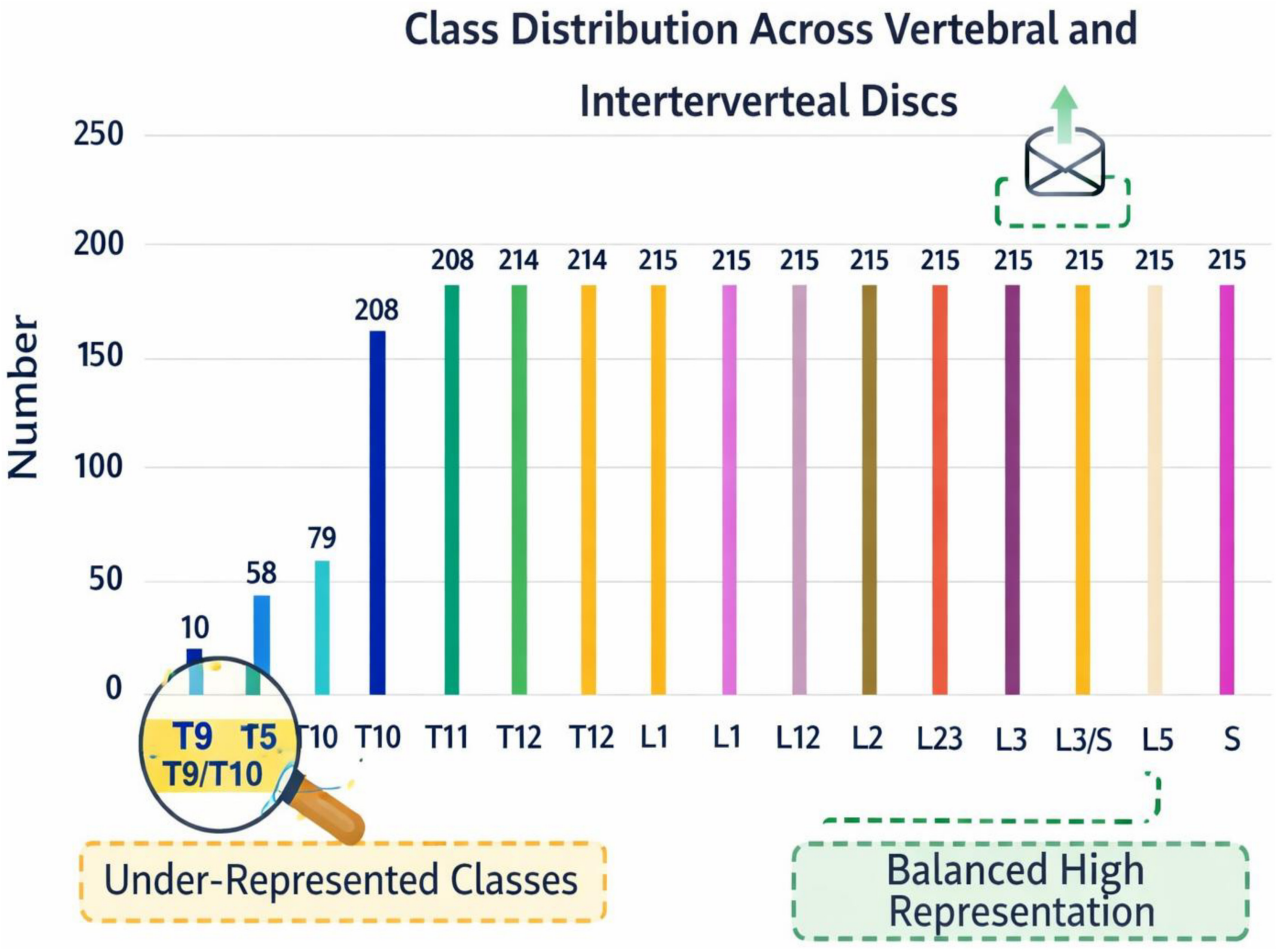
Distribution of annotated spinal structures in the dataset, highlighting class imbalance across vertebrae and intervertebral discs.

The dataset shows different imbalances across the annotated spinal structures, which reflects the classifying features both from an anatomical and clinically prevalence perspective. Even though every subject has the major lumbar vertebrae and the majority has the intervertebral discs, the vertebrae, discs and associated pathologies have substantial differences in the number of corresponding class voxels. More significant vertebrae, especially the L4–L5 and sacral regions, have a significantly bigger proportion of voxels while vertebrae in the upper thoracic area and some vertebrae discs, have less voxels (25). More pathologic changes in degeneration, disc herniation and endplate osteochondritis, differences across the classes also add variability in the shape and volume of the classes. Like as shown in Figure 3, imbalances across vertebrae disc classes makes the frequency of the disc classes look like an elongated tail. More classes concentrate on one end while less classes concentrate on the other end. A lot of the data focuses on a few of the vertebral structures which makes the data more imbalanced. This data imbalance makes it more difficult to learn robust, strong representations. This imbalance can partly explain the lower segmentation accuracy across mid thoracic vertebrae. This is why a graph based semantic approach is used the most. This approach improves segmentation of the less represented classes.

### 3.2 Preprocessing

Before advancing to the 3D coarse segmentation phase, every image underwent the processes of cropping, scaling, padding, and normalization. While cropping the video, the images were centered and trimmed using a DoHoWo2 bounding box. Then, any spine-related missing voxels were removed. After that, images were resized, and the voids at the vanishing points were padded with zeros to reach the target of 18,256,128 voxels. After performing the aforementioned processes, the MR images were normalized by applying the formula (voxel value − voxel mean)/voxel mean (26). For enhancement of the 2D segmentation process, each image slice was also padded and resized to dimensions of 512 by 256 pixels.

Summarizing the preprocessing pipeline for T2-weighted MR images, prior to segmentation, is shown in Table 1. These steps include background area reduction through cropping, spinal structure localization, and padding to make model input sizes consistent. Normalization of voxel intensity is described to keep the overall distribution of the values consistent for all the subjects. The 3D coarse segmentation model will be trained on the data after it is reduced to a lower resolution. After it is segmented, data will be divided into axial slices, and the slices will be resized for 2D refinement at a higher resolution. In terms of data to make the model more robust and generalizable, data augmentation will be applied through variations of rotation and voxel intensity.

**TABLE 1:** Summary of preprocessing steps for the proposed spine segmentation framework.

Step no.	Preprocessing operation	Description	Output resolution/ format
1	Spine localization and cropping	Central cropping using bounding box to remove non-spinal voxels	Spine-only volume
2	Padding	Zero-padding to ensure uniform input size	182 × 256 × 128 voxels
3	Intensity normalization	Mean subtraction and standard deviation scaling	Zero-mean, unit-variance
4	3D volume resizing	Resized for coarse segmentation	18 × 256 × 128
5	2D slice extraction	Axial slices extracted from 3D volume	2D axial slices
6	2D slice resizing	Resized for refinement network	512 × 256 pixels
7	Data augmentation	Rotation (−15° to + 15°), brightness and contrast adjustment	Augmented samples

**FIGURE 4:**
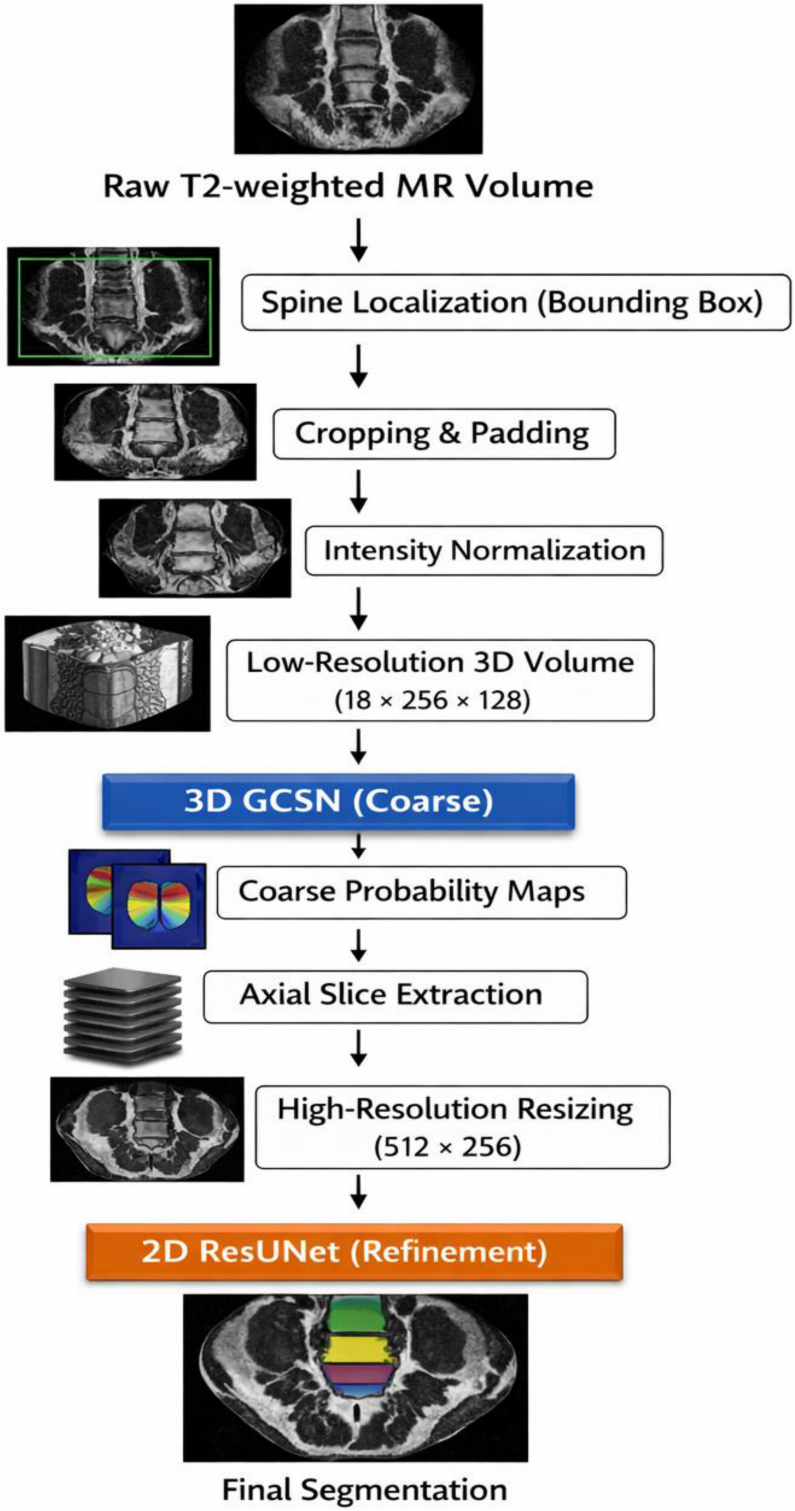
Preprocessing workflow for the proposed spine segmentation framework.

The complete dissecting and reprocessing workflow in the proposed spine parsing framework is showcased in Figure 4. The pipeline starts from raw T2-weighted MR volumes and progresses to spine localization with bounding boxes to hone in on the area of interest. Input sizes are standardized by cropping and padding and subsequently, intensity normalization is carried out to address the varying voxel distributions across the subject volumes. The volume is then downsampled to a 3D low-resolution volume to enable efficient coarse segmentation with the 3D Graph Convolutional Segmentation Network (GCSN). The coarse segmentation probability maps are forwarded to axial slice extraction and then to high-resolution upsampling to capture the finer structures. A 2D ResUNet segmentation refinement network sharpens the boundaries and overlays the final segmentation of the vertebrae and intervertebral discs (27). The two-stage design is effective in the context of global contextual learning coupled with high-resolution local refinement.

### 3.3 Implementation details

Five approaches provided for the 3D coarse segmentation for correctness and robustness of results. The methods evaluated were 3D ER, 3D ResUNet, 3D Graphonomy, 3D GCSN, and a 3D DeepLabv3 + variant. Also, for consideration were the standard 3D GCSN and the DeepLabv3 + based one. For the 3D GCSN architecture, the encoder path had 4 layers and in the first encoding stage there were 32 feature maps. The encoder had a total of 32 layers in operation. 3D ResUNet model (28) had an encoder split into 5 levels. The first encoding stage was also initialized with 32 feature maps. The encoder comprised 33 layers in total. 3D ResUNet and 3D GCSN have similar structures with the exception of the former lacking the semantic extraction module. 3D DeepLabv3 + based GCSN has the same primary architecture as the 3D GCSN, the only difference in the design of the semantic modeling backbone. In the 3D Graphonomy variant, the 3D GCSN architecture (29, 30) substitutes the zone-pooled and provinced components with the matrix transformation mechanism from the initial Graphonomy framework. This change improves the segmentation results’ reliability and accuracy. Other than this substitution, the other phases of the 3D GCSN architecture are in accordance with the layout and processing flow of the 2D GCSN.

### 3.4 The proposed method

Using the method proposed has two primary benefits compared with traditional methods that analyze vertebrae and intervertebral discs (IVDs) separately. First, it can study and split vertebrae and IVDs simultaneously, which is a biomechanical advantage since vertebrae provide structural support to the IVDs. In biomechanical modeling, vertebrae and IVDs lacking overlap is important to maintain even load distribution and biomechanical realism. Second, the proposed method purposely and to the best of its ability uses the semantic relationship between vertebrae and IVDs. This is important in the discrimination of consecutive elements of the spine and accurate and detailed disc and vertebrae numbering. This type of modeling is useful in the downstream biomedical engineering and surgery processes where the vertebrae and IVDs need to be clearly distinguished. This is particularly important for the proposed method’s enhanced classification and modeling specifics.

Most current research on uniting vertebrae and IVD segmentation continues to utilize classical computer vision or deep learning methodologies. Examples include the usage of the radial basis function (RBF) coupled with artificial potential fields and radial basis function (RBF) with the mid and multi-resolution contextual features (19, 20), which achieved the simultaneous segmentation of vertebral bodies (VBs) and intervertebral discs (IVDs) within the MR images of morbidly obese subjects. They achieved a classification accuracy of 92.5% for vertebrae and 91.4% for IVD boundary detection within the 9.2-min time frame of the analysis. Additionally, the multi-modality adversarial networks (31) for neural canal segmentation obtained concurrent segmentation of vertebrae, discs and neural foramina on the MR images of T1 and T2 and obtained DSC (Dice similarity coefficient) values of 92.59% for vertebrae and 88.01% for discs and IVDs. Nevertheless, all these methodologies still provided one label for all vertebrae and one label for all IVDs, making it impossible to distinguish the individual components of the spine.

The new method creates labels for each vertebra and each intervertebral disc (IVD), allowing for a high level of detail in distinguishing different parts of the anatomy. This level of detail is important for the planning of minimally invasive procedures, the analysis of spinal mechanics, and the evaluation of the progression of diseases. The improvement of the receptive field and contextual modeling in convolutional neural networks (CNNs) has been the focus of increasing the level of semantic representation. Increasing the depth of a neural network, use of pooling operations, and the application of residual learning, such as in a ResNet (32), to reduce the performance drop of deep networks, are common methods. Although pooling operations are useful in increasing contextual understanding, they often do so at the expense of the loss of detailed microstructures. This results in the segmentation boundaries being more blurry than they need to be. This problem is solved by U-Net (24) with the use of skip connections. DeepLabv3 + , on the other hand, uses atrous spatial pyramid pooling (ASPP) to further enhance representation at multiple scales and attention-based techniques (33) use focus to improve the differentiation of features to enhance representation at multiple scales.

The use of sequential modeling techniques has also been investigated. An example is Spine-GAN (27) which used long short-term memory (LSTM) units to encode the semantics of the relationships between different parts of the spinal region by using the spatial interdependencies of the feature maps. However, since the LSTM framework was devoid of any explicit anatomical knowledge or spatial constraints of the spinal structures, the segmentation results left much to be desired. This was particularly the case for the vertebrae which were anatomically similar and therefore difficult to differentiate.

Graph-based semantic modeling is an area of significant potential. The human parsing framework Graphonomy (19) involves mapping spatial constructions to graph structures using matrix transformations and graph convolutional operations to encode and capture the semantic relationships. After the relationships of the graph structure are encoded, the graph representation is re-projected into the image space by a series of matrix operations. While Graphonomy captures spatial relationships between anatomical parts and their constituents, both the graph construction and re-projection steps are devoid of any anatomical information, making them opaque and inefficient for the tasks of segmentation that are driven by anatomy.

**FIGURE 5:**
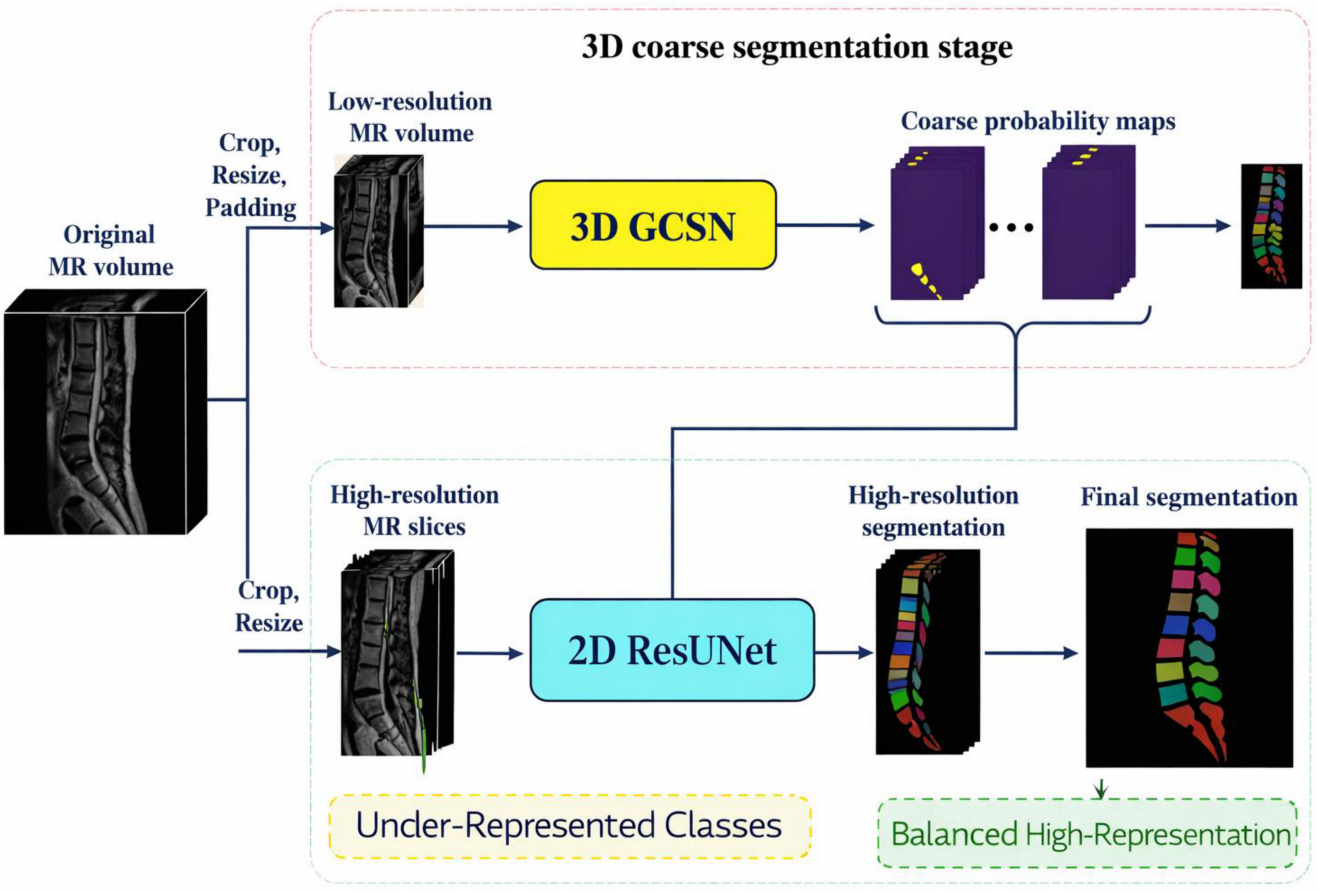
Overview of the proposed spine parsing framework for automated segmentation of vertebrae and intervertebral discs from volumetric MR images.

Inspired by these issues, we design an anatomically directed, memory-efficient spine parsing framework, as shown in Figure 4. The memory-efficient 3D Graph Convolutional Segmentation Network (GCSN) for volumetric MR images recovers semantic representations by modeling spatial and anatomical relationships of the consecutive vertebrae and the IVDs for a series of constrained MR volumes. The design aligns with the responses of human anatomy, thereby reducing intra-class variability and enhancing uniformity across the constituents of the spinal system. The 3D GCSN incorporates region pooling modules that convert volumetric features into graph representations. A meticulously constructed adjacency matrix, as shown in Figure 5, captures the biomechanical and anatomical relationships of the vertebral structure, where each node represents an individual element of the spinal system.

Moreover, the suggested model employs moderate 3D compression, which compared to other volumetric segmentation frameworks, results in significantly less memory consumption while still preserving accurate segmentation. This trade-off between efficiency and performance must be why the proposed methods are ideal for expansive clinical implementations and areas with limited resources.

Figure 5 summarizes the proposed two-stage spine segmentation framework which consists of coarse 3D volumetric segmentation and fine-grained 2D slice-wise refinement. In the first stage, the original MR volume is preprocessed by cropping, resizing, and padding and then the system removes the non-spinal areas and produces a low-resolution MR volume. The 3D Graph Convolutional Segmentation Network (3D GCSN) then processes this volume. The 3D GCSN exploits volumetric context and graph-based semantic relationships among the vertebrae and intervertebral discs (IVDs). The 3D GCSN creates coarse probability maps for each spinal part, and while it captures the global anatomical structure, it has limited boundary precision due to the initial downsampling. In the second stage, the original volume is used to extract high-resolution MR slices, which are then paired with the coarse probability maps created by the 3D GCSN. A 2D ResUNet boundary refinement and fine detail restoration are added to the fused inputs (34). In this stage, the information loss due to 3D downsampling is addressed, and segmentation accuracy is increased, especially for IVDs and other small structures that are poorly represented. The end result is a high-resolution, multi-class segmentation with clear separation of vertebrae and IVDs, sharp delineation of boundaries, and mitigation of class imbalance. The framework evolves from coarse segmentation of underrepresented classes to the final output, which is balanced and well-defined.

**FIGURE 6:**
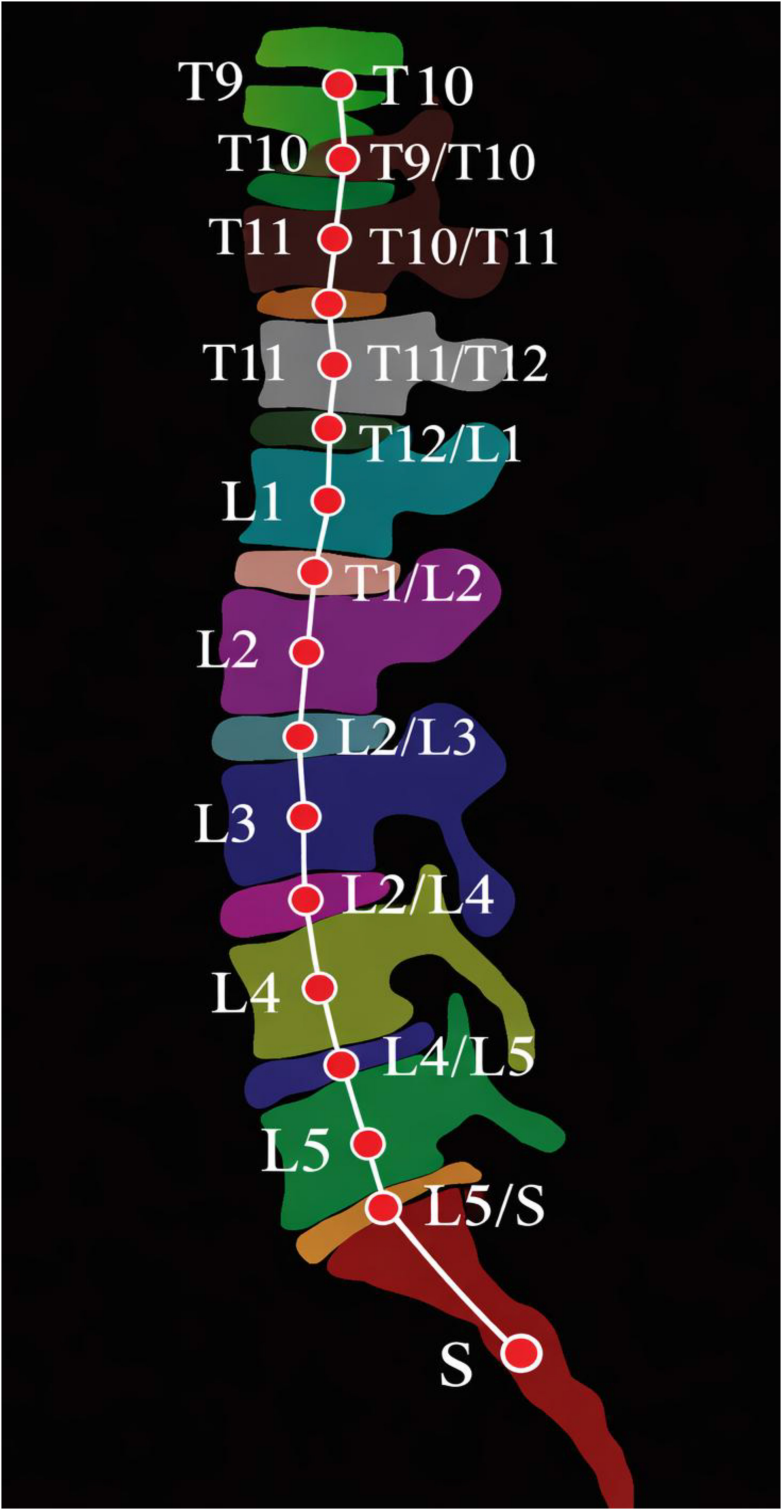
Adjacency matrix encoding anatomical relationships between vertebrae and intervertebral discs used for graph-based semantic modeling.

One of the most obvious anatomical relationships in the spine is the adjacency of vertebrae. Figure 6, a white line between two nodes indicates a connection of some sort between the two spinal structures. Among many other reasons, factor C is important for modeling the inter-vertebral relationship. This study makes several contributions, reported here in no particular order. The first contribution is the design of an automated spine parsing framework for volumetric MR images that can simultaneously and uniquely identify and segment individual vertebrae and intervertebral discs (IVDs) (35). This method provides the potential for accurate cross-separation of spinal structures from volumetric MR datasets. The method may assist in the formation of clinical applications for the biomechanical assessment of the spine and the diagnosis of spinal disorders, including intervertebral disc degeneration and radiofrequency ablation (RFA)—related disorders.

Second, we articulate an innovative segmentation framework, tailored for segmented MR volumetric data for columns. This framework consists of two networks working in tandem: a 3D coarse network segmentation for low-resolution volumes, and a 2D network for refining segmentation on high-resolution axial slices. These networks are designed to tightly couple for optimal integration and collaboration (19, 34). This approach mitigates the issues of processing full-resolution 3D volumes and allows for the use of standard desktop computers, which enhances the framework’s accessibility in clinic environments.

Third, the use of graph convolutional network (GCN) in the proposed framework captures the relationships among the components of the spinal column. This explains the abilities of the model in attempting solving issues due to structural resemblance, variation between the subjects, and the anatomy being heterogeneous. The proposed method of reasoning about the structure of the spinal column connected this way, clarifies and sorts out the complicated anatomy and enhances the differentiation of closely associated vertebrae and IVDs. Figure 6 depicts the overall design of the framework, specifically demonstrating the coupling of the 3D Graph Convolutional Segmentation Network (GCSN) with the 2D ResUNet. As illustrated, the 3D GCSN produces coarse probability maps based on the low-resolution MR volume. Even though the segmentations are coarse, they are the only ones that provide sufficient global context. With the help of the 2D ResUNet, these maps (along with the MR slices of corresponding higher resolutions) have their boundaries refined and their anatomical details restored. This method effectively reduces the information loss that is typically caused by downsampling in 3D. Overall, it allows for accurate boundary tracing and boundary loss in the final segmentation.

The 3D Generalized Convolutional Spherical Neural Network (GCSN), shown in Figure 6, incorporates a deep convolutional backbone, a semantic feature extractor, and a specialized module that converts conventional CNN feature maps into structurally aware representations by explicitly modeling the spatial relationships among spinal components, including vertebrae and intervertebral discs (IVDs). Unlike most convolutional networks that focus predominantly on local appearance cues, the GCSN also utilizes additional context, such as anatomical adjacency and spatial order, to differentiate visually analogous components from one another based on the spatial configuration of their relationships (36). The figure illustrates this concept effectively. The deep convolutional network handles the primary input image and the auxiliary input representation simultaneously. The semantic feature extractor and the decoder, together, control the formation of the high-level semantic representations and the construction of initial coarse probability maps. Additionally, the use of residual connections integrates low-level feature representations from the shallow convolutional layers with higher-level semantic features. These residual connections, as shown in the figure, streamline the passage of information and retain critical information for the segmentation process, which enhances the quality of the segmentation.

The 3D Generalized Convolutional Spherical Neural Network (GCSN), shown in Figure 6, consists of a deep convolutional backbone, a semantic feature extractor, and a special module that converts conventional CNN feature maps into a structure-aware feature Map. This module explicitly spatially relates the features of the spinal anatomy components, i.e., vertebrae and intervertebral discs (IVDs). While most convolutions spatially relate the features of a single component, the GCSN utilizes spatial relationships of multiple components in its construction, as GCSN offers spatially different contextual cues like anatomical adjacency and relative positioning, which are not present in traditional Convolutions (37, 38). The figure shows this idea effectively and intuitively. The deep convolutional network is responsible for the processing of the primary input image and the auxiliary input representation. Along with the decoder, the semantic feature extractor serves to create the high semantic features and the early probability maps (which are of lower resolutions). Furthermore, residual connections are utilized to connect the features of lower levels, which are produced by the convolutional layers, to the higher semantic features. These connections, which are described in the figure, showcase outstanding information flow and keep the micro details of the image from being discarded, which boosts the quality of the segmentation.

**FIGURE 7:**
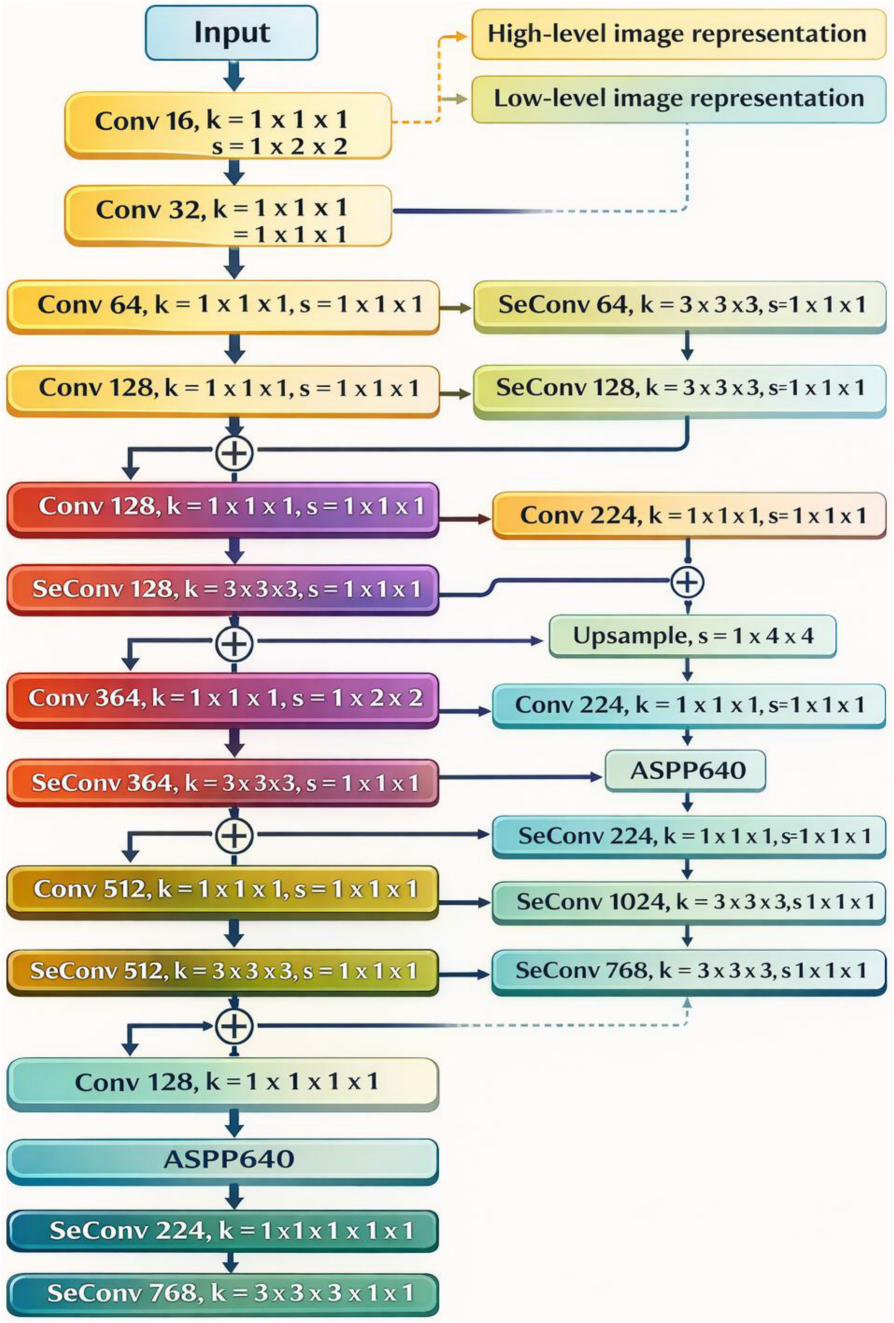
Architecture of the proposed 3D graph convolutional segmentation network (GCSN) for coarse volumetric spine segmentation.

The first part of the framework is a deep convolution neural network made of different specialized modules. A depthwise convolution layer is used to represent the different levels and dimensions of the images. Following the DeepLabv3 + framework, this constructed depthwise convolution layer aims to achieve more precise feature encoding and to preserve loss, as noted in Figure 7, to preserve the reliability of feature extraction. This design choice, in Kipf and Welling (39) reduces the amount of computation needed by bypassing unnecessary propagation of features and still preserving a high representational complexity. This means that the network is built to accommodate more difficult segmentation challenges. Starting point of the processing pipeline is creation of a compact visual model of the spinal column from the source MR images. This condensed visual model is used for further feature extraction and semantic analysis aimed at capturing as much anatomically relevant detail as possible from the spinal region.

**FIGURE 8:**
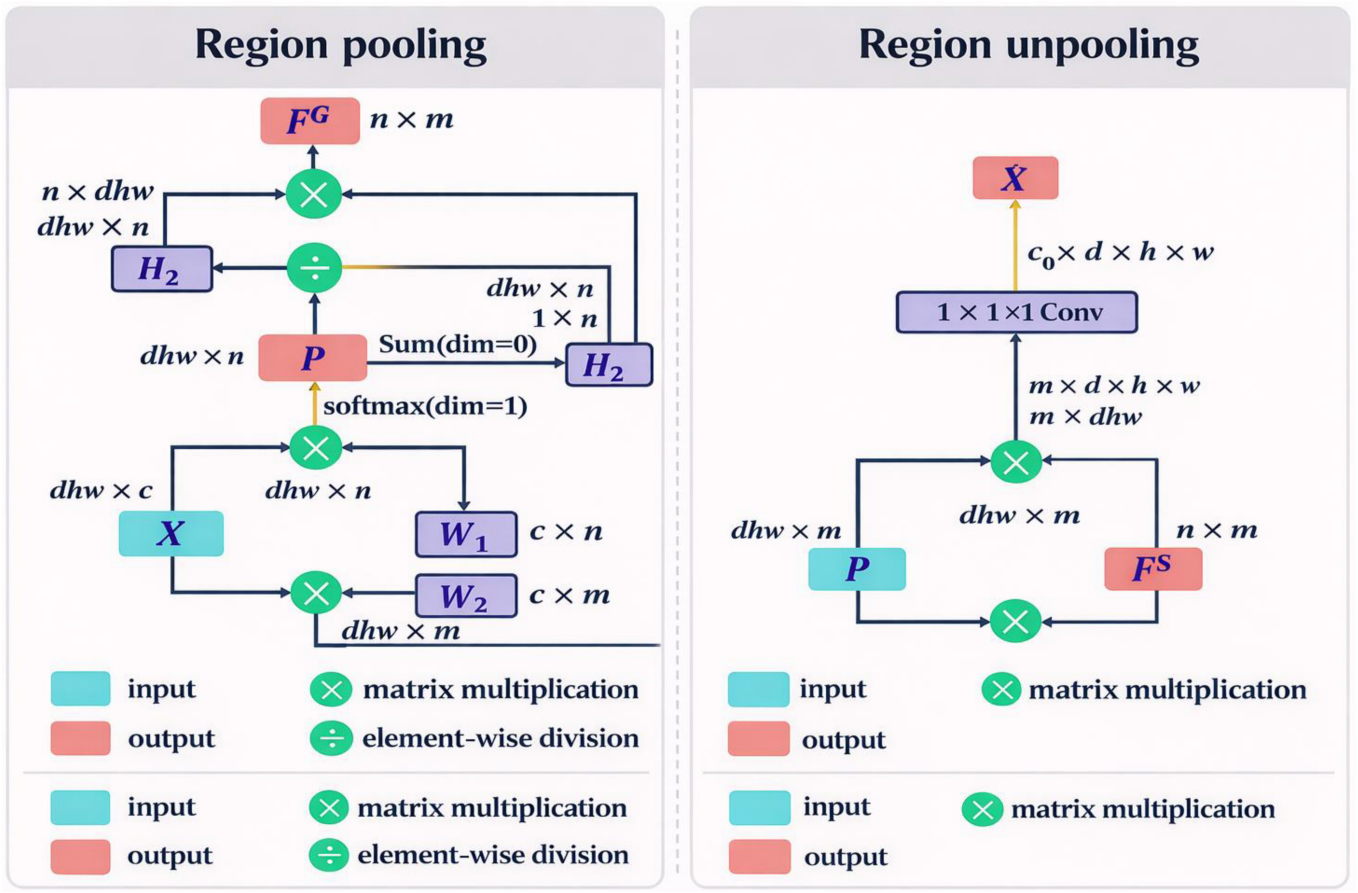
Internal architecture of the proposed two-stage segmentation framework integrating 3D GCSN and 2D ResUNet refinement.

Figure 8 illustrates the hierarchical architectural framework of the 2D ResUNet. The 2D ResUNet operates by jointly utilizing the corresponding coarse probability map slice and the high-resolution 2D axial MR image. The network produces *n* output streams, each responsible for predicting a specific subcategory, and these streams collectively form the final multi-class segmentation output. The fusion of these streams is achieved through shared weight vectors and a multi-class bridging mechanism that is intrinsic to the 2D ResUNet architecture. By effectively integrating contextual information with the distinctive structural characteristics of vertebrae and intervertebral discs, the 2D ResUNet is able to generate higher-quality segmentation results. The availability of coarse probability maps generated by the 3D GCSN enables the 2D ResUNet to indirectly leverage 3D semantic information during refinement. These probability maps encode volumetric contextual cues derived from the 3D MR data and serve as an additional guidance mechanism during 2D segmentation. Furthermore, the high-resolution axial MR slices provide fine-grained spatial details that complement the volumetric semantics. By fusing 3D semantic knowledge with detailed 2D feature representations, the 2D ResUNet refines the segmentation boundaries and produces a more accurate and anatomically consistent representation of the spinal structures.

**FIGURE 9:**
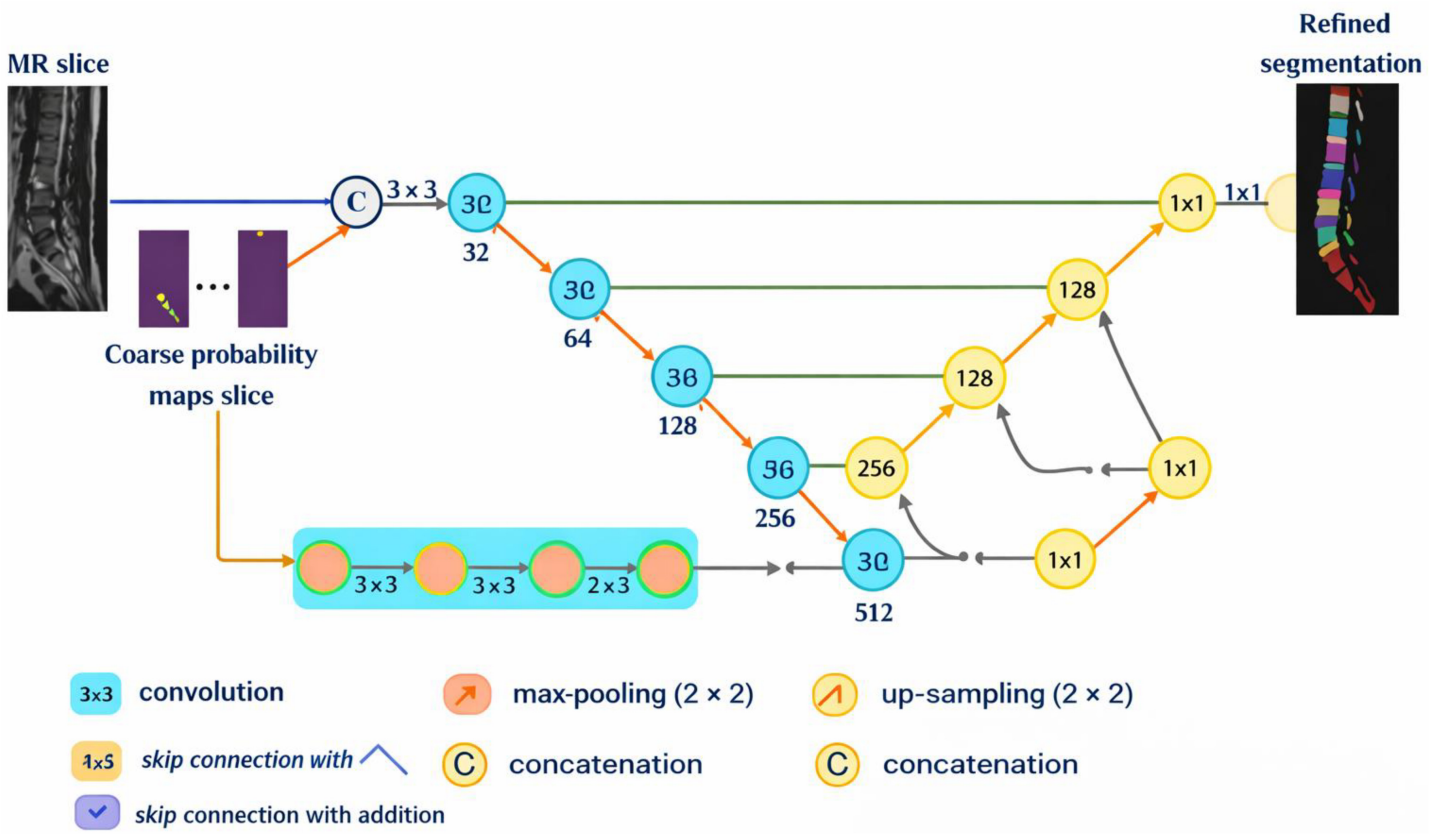
2D ResUNet-based refinement process for enhancing boundary accuracy using coarse probability maps and high-resolution MR slices.

The framework’s first element involves a Deep Convolutional Neural Network (DCNN) architecture built with a set of customized modules. A specific depthwise convolution layer is incorporated and serves to represent both low and high image representations. Similar to the DeepLabv3 + framework this depthwise convolution layer is built for better feature encoding with less loss and more retrieval of the features as shown in Figure 9, thus improving the reliability of the selected features. This design choice not only eliminates redundant intermediate features but also provides in a high representational capacity. With fewer features in the construction, the network is left with more freedom to tackle harder segmentation problems. The first step in the processing pipeline involves creating a dense visual representation of the spinal column based on the input MR images. This representation is also used to trigger the next steps of feature extraction and the spinal region semantic analysis, aiming to effectively capture and preserve meaning of the anatomical details.

## 4 Results and analysis

### 4.1 Overall performance

All quantitative results indicate the average standard per structure segmentation evaluation metric for the entire cohort. More specifically, the Dice Similarity Coefficient (DSC, %) is the percentage of spatial overlap between the predicted segmented output and the ground truth, precision (%) is the percentage of correct predictions out of the total number of predicted voxels belonging to a class, and recall (%) is the percentage of the ground truth voxels that were captured. The mean and standard deviation of all values are reported to reflect the overall accuracy and the distribution of results. The established baselines are the proposed methods along with the more established pure 3D architectures (3D U-Net, 3D ResUNet, 3D DeepLabv3 + , and 3D Graphonomy) and hybrid 3D–2D frameworks that incorporate volumetric segmentation and slice-wise refinement. These methods are considered to be the state of the art for vertebrae and intervertebral disc segmentation. Despite the baselines, metrics indicate that the proposed graph-aware two-stage framework achieves the most competitive mean DSC with the least standard deviation for the vertebrae, intervertebral discs, and total of 19 spinal structures.

The most significant performance improvements are seen in the most difficult regions encountered, hinting the most challenging areas with high inter-class similarity. The results show that, while the proposed method most definitely utilizes high-resolution 2D refinement and the best current methods in 3D global context, it results in the highest accuracy and consistency.

**FIGURE 10:**
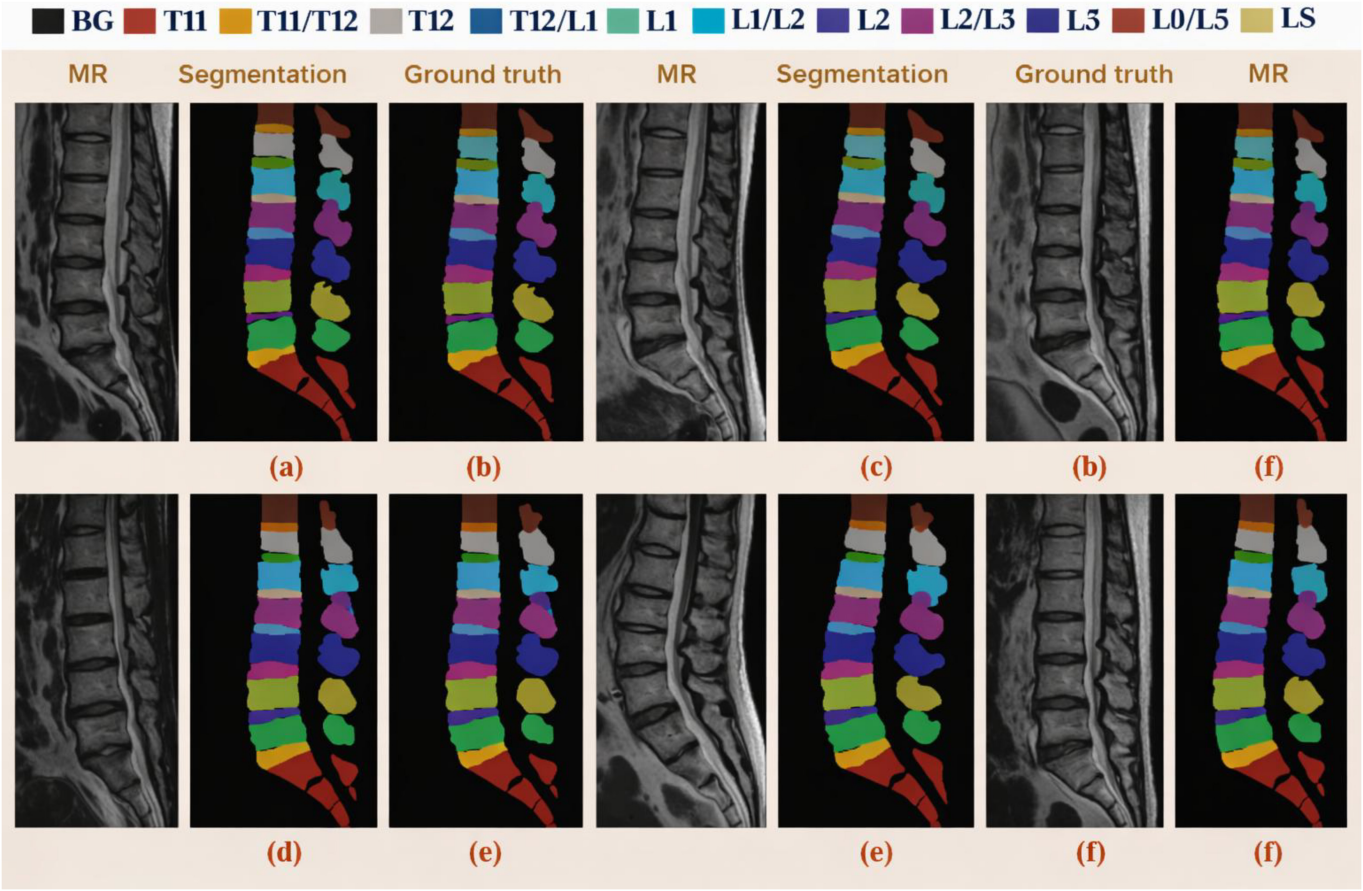
Examples of segmented MR images of lumbar spine visualized qualitatively. **(a)** Predicted segmentation superimposed with Case 1’s sagittal MR image. **(b)** Case 1’s corresponding expert-annotated ground truth. **(c)** Robustness to anatomical variation is demonstrated by predicted segmentation on a 2nd MR image. **(d)** Predicted segmentation on a 3rd MR scan with consistent labeling across the lumbar and sacral regions. **(e)** Ground truth annotation corresponding to panel **(d)**. **(f)** Segmented prediction on yet another MR image demonstrating the framework’s stability and generalizability across various imaging conditions.

To Figures 9, 10 show the segmentation results for the vertebrae and spinal structures. The analysis provided in the following paragraphs focuses on these figures. The results speak for themselves and the high accuracy of the segmentation boundaries can be attributed to the two-stage segmentation framework which maintains and fine-tunes the boundaries of the segments. The average model recall for vertebrae segmentation is 88.00 ± 6.44% while intervertebral discs (IVDs) is 90.43 ± 5.55% and for 19 vertebrae components is 89.09 ± 5.20%. There are slightly more segmentation falses than the overs and the mean precision values show just under the recall values which averages to 87.30 ± 5.54% for vertebrae, 86.58 ± 3.80% for the IVDs, and 85.86 ± 4.84% for all components. Achieving the proposed model means DSC for all 19 components is 87.49 ± 3.81%, for vertebrae is 87.32 ± 4.75% and for IVDs is 87.78 ± 4.64%. The segmentation approach is robust and accurate based on the comparative grounding truth.

**FIGURE 11:**
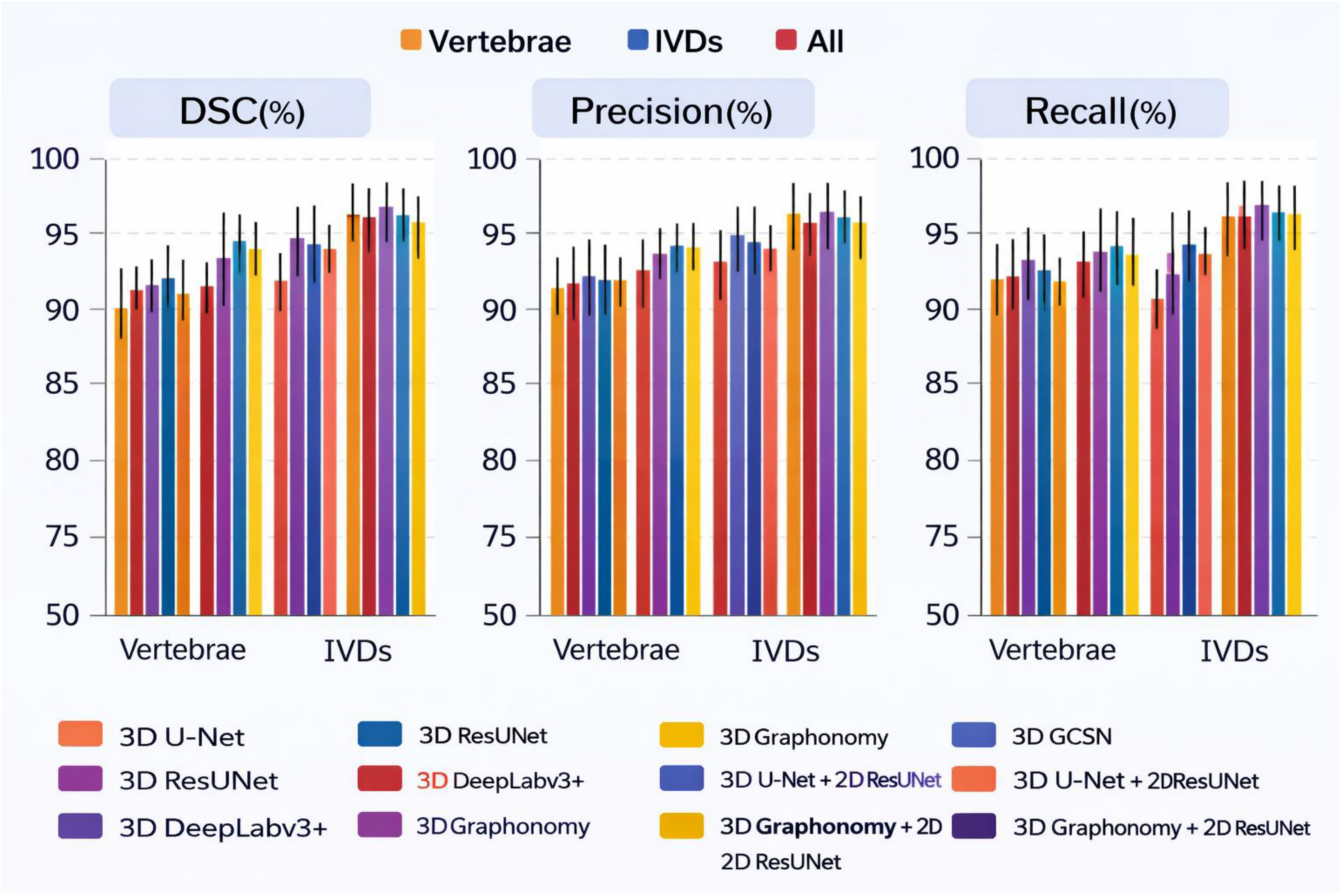
Quantitative comparison of mean dice similarity coefficient (DSC) and precision across 19 spinal structures for different methods.

This seems to be the only suitable context for showing the results from various spinal MRI segmentation methods evaluated with the Dice Similarity Coefficient (DSC), Precision, and Recall for each of the vertebrae, intervertebral discs (IVDs), and overall spine components shown in Figure 11. Regarding left panel segmentation accuracy, all Pure 3D Models (3D U-Net, 3D ResUNet, 3D DeepLab3 + , and 3D Graphonomy) are more or less about the same. However, they are less accurate than the rest of the hybrid models. Of all the models, the 3D coarse segmentation/2D ResUNet refinement models are the most respectable. The 3D models, in particular, appear to be on the poorer end of the spectrum or somewhere in the mid-range, particularly in ground truth overlap for the IVDs. Middle Panel: Precision: Segmenting Techniques on Vertebrae. Most techniques perform similarly. Hybrid 3D-2D models show refined IVDs. This is especially true for the combination of all three models, which indicates improved boundary detection and less overshooting. Right Panel: Recall: Proposed Hybrid models with the most IVDs = Highest Recall, indicating they capture the most of the entire anatomical structure. Across the models, there is a tendency to segment conservatively by including all the parts that were not overshot, suggesting a conservative segmentation style. Overall, the figure displays that the combination of graph-aware 3D modeling and 2D boundary refinement offers the best trade-off and the most consistent result across all evaluation metrics. The less varianced error bars illustrate an increased consistency of the proposed method.

In Table 2, T9 through S1 vertebrae level individual mean Dice Similarity Coefficient (DSC, %) values are compared among various segmentation techniques. Most techniques are less accurate in the upper thoracic (T9–T11) due to smaller anatomy and more variability between subjects, and more accurate in the lumbar and sacral areas. The baseline techniques are less robust and more variable compared to the proposed method, which demonstrates high and consistent DSC values across the lumbar vertebrae and S1.

**TABLE 2:** Anatomically resolved comparison of vertebral segmentation accuracy.

Method	T9 vertebra	T10 vertebra	T11 vertebra	T12 vertebra	L1 vertebra	L2 vertebra	L3 vertebra	L4 vertebra	L5 vertebra	Sacrum (S1)
3D GCSN	29.64 ± 2.01	61.58 ± 8.30	79.79 ± 1.33	88.77 ± 6.84	86.37 ± 2.42	88.18 ± 2.64	85.68 ± 2.68	86.67 ± 2.84	84.25 ± 2.57	86.25 ± 2.40
3D U-Net + 2D ResUNet	26.13 ± 3.77	40.91 ± 4.18	78.84 ± 2.88	82.26 ± 2.10	83.54 ± 2.96	83.64 ± 9.44	84.32 ± 8.28	86.91 ± 4.02	89.32 ± 7.77	87.92 ± 3.29
3D ResUNet + 2D ResUNet	28.43 ± 2.09	53.25 ± 0.69	85.38 ± 9.55	88.15 ± 4.77	85.70 ± 9.32	89.54 ± 1.13	89.01 ± 7.18	89.86 ± 7.19	89.93 ± 6.78	89.01 ± 4.81
3D DeepLabv3 + + 2D ResUNet	38.58 ± 0.39	67.44 ± 8.19	93.85 ± 4.40	86.78 ± 1.79	87.33 ± 7.84	87.23 ± 0.44	87.13 ± 1.33	87.38 ± 0.29	86.65 ± 6.83	86.82 ± 2.87
3D Graphonomy + 2D ResUNet	49.51 ± 32.92	56.65 ± 38.12	81.87 ± 22.72	88.94 ± 9.71	90.10 ± 6.94	89.20 ± 7.73	89.24 ± 6.16	89.13 ± 5.89	88.97 ± 6.54	88.89 ± 3.04
Proposed method	36.62 ± 38.52	60.15 ± 36.62	82.63 ± 21.00	89.53 ± 6.69	90.70 ± 2.63	90.08 ± 2.58	89.87 ± 2.66	89.64 ± 2.69	89.63 ± 2.35	89.22 ± 2.18

**FIGURE 12:**
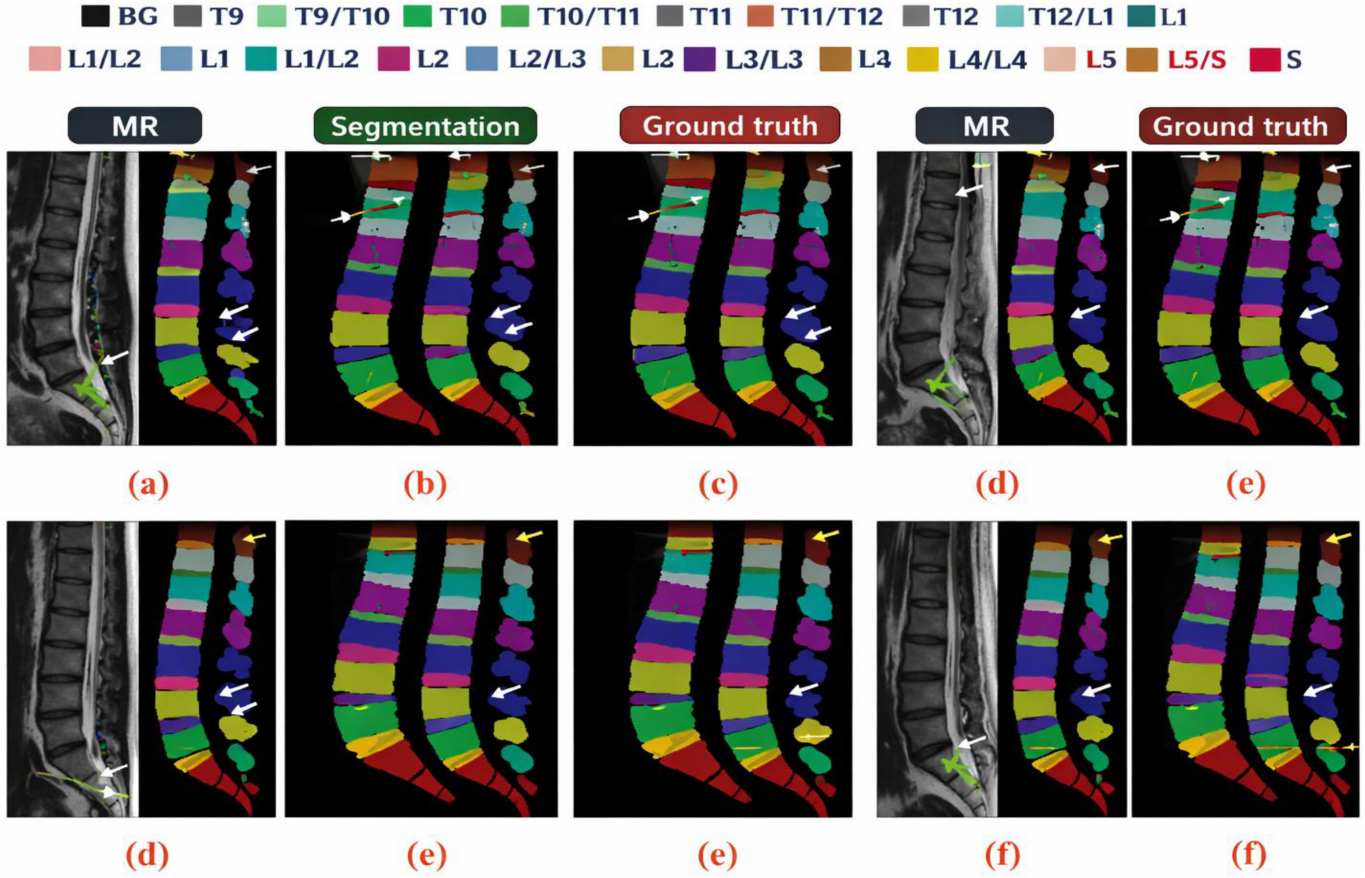
Qualitative comparison of lumbar spine MR images and segmentation results. **(a)** Original MR image. **(b)** Predicted segmentation. **(c)** Corresponding ground truth. **(d)** Additional MR case. **(e)** Predicted segmentation for panel **(d)**. **(f)** Ground truth annotation, demonstrating strong agreement and robustness of the proposed method across challenging regions.

It must be noted how each element contributes to the overall outcome. The restricted generalizability of the proposed model is illustrated in Figure 12. The absence of the T9–T11 vertebrae and IVDs challenges segmentation due to the differing morphologies of the surrounding bones. Consequently, this increased difficulty in targeting specific bones remains. Based on the upper portion of the image, the relative lack of T9–T11 pixels results in a smaller input T9–T11 image patch, which is proportional to the spatial position of the image patch, is a contributing factor to the loss of semantics. Because most of the T9–T11 pixels are in the upper part of the image, there is a loss of semantic information. The result is that 3D GCSN can outperform all other methods in terms of mean DSC when segmenting each of the vertebrae and IVDs. Figure 11 illustrates that 3D GCSN achieves a DSC that is 1.59 points higher than that of 3D DeepLabv3 + for the segmentation of all 19 spinal structures (85.97\10.71%). This is particularly evident when one contrasts the conventional approach with 3D DeepLabv3 + . When comparing to the earlier method of 3D DeepLabv3 + , this is evident. When the two numbers are considered, this is clearly the case. The most straightforward justification for the improvement in parsing performance due to the addition of a semantic feature extractor to Spine is the extractor itself being implemented. Spine parsers’ performance may improve because of the semantic feature extractor, particularly in patients with large S1/S2 gaps. The 3D DeepLabv3 + model is unable to identify S1, L5, and IVD, even when those structures are indistinguishable, due to the absence of a certain semantic attribute. This is true even when the IVD is the same as S1. The lack of sensitivity of the 2D ResUNet model to vertebral and intervertebral disc (IVD) structures is responsible for poor data segmentation. Resultantly, unnecessary ambiguity is added, making the problem more difficult to solve. The semantic feature extractor is used in 3D GCSN to create a semantic representation of the image. This is also important because the image demonstrates the complex relationship between different parts of the spine and vertebral, as well as the intravertebral disc, structures. This is important because of the relationship between the spinal components. Indeed, this explains most of why the 3D GCSN has better performance compared to the 3D DeepLabv3 + . Unlike other methods, the new approach uses a non-linear function, but only at a separate stage. This is possible because of the reduced number of adjustable parameters. With the training data set being smaller, the network won’t require as much memory, and in turn, will have a better generalization capability.

### 4.2 Statistical significance analysis

In order to determine the statistical significance of the noted performance gains, paired statistical tests were performed on the Dice Similarity Coefficient (DSC) across all individual subjects. A paired *t*-test was conducted to assess each proposed method against each baseline model for each vertebral and intervertebral disc class. A 95% confidence interval for *p* < 0.05 was deemed to be statistically significant. The proposed method has, for the most part, statistically significant gains over most of the competing approaches across the majority of spinal structures.

Table 3 carries out an analysis of statistical significance of the proposed method of segmentation in comparison with the three baselines using the Dice Similarity Coefficient (DSC). To gauge if the differences in performances among the test samples are attributable to chance, a paired *t*-test was conducted. The proposed method shows a statistically significant improvement over 3D U-Net and Graphonomy with a *p*-value of less than 0.01 (strong significance), and less than 0.05 over DeepLab v3 + (moderate significance). These findings substantiate that the enhancements in performance are statistically valid in the proposed method and are not attributable to random occurrence.

**TABLE 3:** Statistical validation of segmentation performance improvements.

Comparison	Metric	Test	*p*-value
Proposed vs. 3D U-Net	Dice	Paired *t*-test	< 0.01
Proposed vs. DeepLabv3+	Dice	Paired *t*-test	< 0.05
Proposed vs. Graphonomy	Dice	Paired *t*-test	< 0.01

## 5 Discussion

With GCN, it appears that it is now easier to differentiate the several spinal components. Confusion matrices take the classification of a voxel approach. Given the acquired conceptual constituent for differentiating several spinal systems, and the vague boundary here between knowledge and vertebral components, the proposed model tends to marginally lose track of adjacent spinal structures aside from the spinal structures and the background. The first projection which is derived from the images used as input to the graph representation and the later re-projection, the technique proposed in this paper would simplify it even further. One is bound to get an advantage in their workflow with the inclusion of this technique. Given that there is a small amount of data available, the signal decoding from the two graphonomy applications is likely to be challenging. For this reason, the available data is scarce. In our experiments, I used both the regional unpooling module and the sector pooled to derive these forecasts, and it was because of this I was able to present them as I did.

At this stage, we experimented with comparing the representation of the images to the networks as we focused on area pooling, dense features from the CNN being converted to a compressed graph representation as a certain node summarizes the details from the features of a particular spinal structure. At this stage, a node feature for each class/node in the graph is created, consolidating the pixel/voxel characteristics from the areas corresponding to a particular anatomical structure. Such a dense representation helps graph convolution layers to do relational reasoning with efficiency. We did this in part so we could figure out the differences between the two, and this work was completed. This is the case because two distinct representations of the same information exist. Consequently, the results were different. An average of the individual voxel’s contributes was used to determine if that voxel’s info should be deemed important. This is the analysis regarding this particular voxel. Concerning the area unpooling method, after the graph convolution modifies node features based on anatomical adjacency, area unpooling reassigns the modified node information back to the voxel grid. Each voxel is imparted a new semantic feature from the connected node(s). The method produces dense feature maps which makes decoding and predicting the probability maps easier. A voxel is typically represented by averaging the representations of component nodes. As the surface area of the voxel increases, the representation of each constituent node increases. The representation of each node is proportional to the chances of the voxel being assigned to the class of that particular node. Taking into account the deep supervision loss, we can estimate the chances of a voxel belonging to each class.

Even with the proposed method’s promise, there are shortcomings to address. Most importantly, while the accuracy of the segmentation improves with the spinal region of the vertebra (T9–T11), there is more size variability, and the training set is underrepresented. All of these cause confusion and a lack of semantic consistency due to vertebral classes. The presence of severe degenerative changes, vertebral deformities, and reduced image contrast may also cause issues where even expert annotators fail to see the borders between vertebrae and intervertebral discs. In such cases, coarse 3D segmentation may also perpetuate minor localization errors to the refinement stage, where the 2D network is unable to retrieve any of the fine boundaries. Because the proposed framework employs a graph-based semantic modeling technique, with a high degree of anatomical accuracy, these comments describe difficulties that represent the extremes of data deficiency and pathological extremes simultaneously. In such cases, the goal of avoiding them will be pursued with the employment of targeted data diversification, pathology-informed training methodologies, and adaptive graph frameworks. Even though the proposed technique shows good results, some shortcomings are noted. The greatest of these is that it exhibits the least segmentation accuracy in the upper thoracic area T9–T11. The vertebrae are smaller, have higher intersubject anatomical variability, and are poorly represented in the training data. This results in lower inter-vertebral confusion semantic consistency, and confusion among the vertebral class adjacencies. Other types of failures arise where test subjects have significant degenerative changes, vertebral abnormalities, or low image contrast. Even expert annotators cannot see the boundaries of vertebrae and intervertebral discs. In this regard, 3D coarse segmentation will during the segmentation process, propagate small localization errors to the refinement step, and the 2D network will be restricted in recovering fine boundaries. The imposition of anatomical fidelity in the proposed framework using graph-based semantic modeling addresses several of these issues, though data sparsity and the extremes of pathology will remain significant concerns for subsequent analyses. More targeted data augmentation techniques, pathology-specific training pathways, and adaptive graph structures are predicted to be more effective in addressing the concerns articulated above.

Even though there are strong quantitative and qualitative results with the proposed framework, some limitations need to be acknowledged. The first is the dataset having an imbalanced representation of the upper thoracic vertebrae (T9–T11) vertebrae. This leads to a decrease of segmentation accuracy and an increase in variability of those regions, as opposed to lumbar and sacral levels. The second is relying on T2-weighted MR images from a single, public dataset. This may affect generalizability of results since there are variances in scanners, imaging protocols, institutions, and patient populations. The third challenge comes from the targeted images having an absence of degenerative changes. The vertebral morphologies are also sometimes abnormal, and the images may have low contrast. localization errors that are still considered minor with respect to the coarse 3D segmentation stage often propagate through to the 2D refinement stage, boundary recovery is also difficult, wherein there is no 2D refinement stage. Lastly, there is a lack of constructive feedback that comes with evaluating the framework in a clinical real-time workflow, and in a multi-center context. The framework also requires further investigation as to its efficacy on large, heterogeneous datasets. Multi-site validation, pathology-aware training strategies, adaptive graph structures, and broader modality testing are ways to address these challenges and set the trajectory for future work.

## 6 Conclusion

This study proposed a unique vertically guided 2-stage deep learning framework for the automated segmentation of vertebrae and intervertebral discs from volumetric T2-weighted MR images. The primary contributions are the coherent modeling of anatomical dependencies using a graph-based approach and semantic learning, the integration of a 3D Graph Convolutional Segmentation Network with the efficient memory architecture for global structure aware segmentation (along with the high-resolution 2D ResUNet for boundary refinement), and the attainment of anatomically resolved multi-class segmentation of individual vertebrae and discs. Numerous qualitative and quantitative analyses show that the proposed systems outperforms the cutting-edge baselines in terms of accuracy, robustness, and anatomical consistency, especially in difficult areas with high inter-class resemblance. The framework is of great value clinically for unmanageable spine parsing for the computer-aided diagnosis, treatment planning, longitudinal monitoring of the disease, and image-guided interventions, and is, from a pragmatic standpoint, sufficiently economical in low and high scale deployed resource demanding clinical settings. In conclusion, this advances the spine segmentation in the state of the art and lays a scalable groundwork for the future clinically deployable systems. state and the art and systems clinically deployable of the future for a groundwork scalable a lays segmentation spine the of state the in art the advances and systems image analysis aware anatomy medical deployable clinically future for a groundwork scalable a lays segmentation spine the of state the in the art the advances and systems image analysis anatomy aware clinically deployable of the future scalable foundation for the state of the art spine segmentation systems. This research opens up a variety of avenues for further exploration. Extending this framework to be more robust and clinically applicable would mean considering multi-center and multi-vendor datasets, as well as incorporating variability in scanner types, imaging protocols, and patient demographics. Second, using a combination of T1-weighted, T2-weighted, and CT imaging, as well as cross-modality learning, could improve segmentation accuracy, especially in areas with low contrast or advanced degeneration, in conjunction with multi-modal imaging. Third, enhanced adaptive and pathology-aware graph structures, which more flexibly adjust their internal anatomical relationships, may better account for uncommon anatomical changes, post-operative spines, advanced deformities, and anatomical gaps. Fostering the integration of uncertainty estimation and explainable AI would improve the clinical trust equation by providing confidence in their segmentation outputs and reasoning for the clinically significant gaps. Finally, ensuring the framework can be optimized for real-time inference will be an important step for clinical deployment and attaining a wider translational impact, particularly when integrated with end-to-end clinical workflows, including image-guided interventions and monitoring for longitudinal changes in disease.

## Data Availability

A Publicly available datasets were analyzed in this study. This data can be found here: The dataset used for the findings is publicly available at the public repository. SPIDER—Lumbar spine segmentation in MR images: a dataset and a public benchmark (Data set). Zenodo. https://doi.org/10.5281/zenodo.8009680. Also available at: https://zenodo.org/records/8009680.
